# COVID-19 Related Arterial Coagulopathy

**DOI:** 10.7759/cureus.9490

**Published:** 2020-07-31

**Authors:** Gagandeep Singh, Hassan Bin Attique, Naga Vaishnavi Gadela, Khubaib Mapara, Srimathi Manickaratnam

**Affiliations:** 1 Internal Medicine, Trinity Health of New England, Hartford, USA; 2 Internal Medicine, University of Connecticut School of Medicine, Farmington, USA; 3 Internal Medicine, University of Connecticut, Farmington, USA; 4 Vascular Surgery, Trinity Health of New England, Hartford, USA

**Keywords:** coronavirus disease of 2019 (covid-19), coagulopathy, arterial thrombosis, venous thromboembolism (vte), acute limb ischemia, left ventricle (lv) thrombus, ischemic stroke

## Abstract

Coronavirus disease of 2019 (COVID-19) is a disease caused by the novel coronavirus SARS-CoV-2, which is characterized by a multitude of clinical abnormalities, including hypercoagulability. Although thrombosis is commonly observed in sepsis, the hypercoagulable state associated with COVID-19 is much more dramatic and may not be related to either the severity of the disease or the D-dimer levels. It may be due to a prothrombotic state induced by the disease itself. We report three cases of arterial thrombosis with a significant clot burden requiring urgent medical and surgical intervention. It is now a common practice to initiate anticoagulation for deep venous thrombosis (DVT) prophylaxis based on the D-dimer level in hospitalized patients with COVID-19. However, in our clinical experience, D-dimer levels did not correlate well with the clot burden or the risk for future thrombosis.

## Introduction

Coronavirus disease of 2019 (COVID-19) is characterized by a multitude of clinical abnormalities, including hypercoagulability [1]. Although thrombosis is common in sepsis, the hypercoagulability associated with COVID-19 may be due to a prothrombotic state induced by the disease itself. We report three cases of arterial thrombosis requiring urgent intervention.

## Case presentation

Case 1

**Figure 1:**
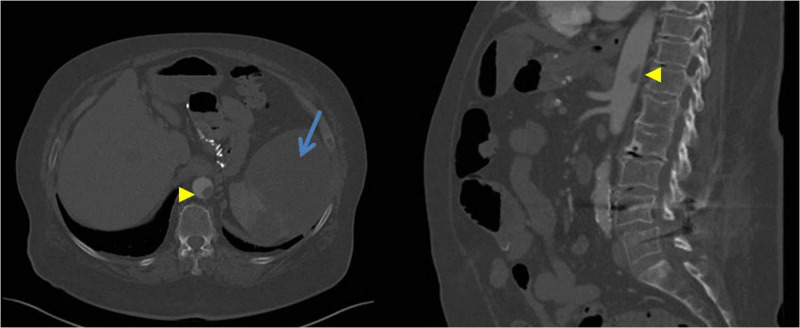
Intraluminal aortic thrombus in the supraceliac and visceral aorta (arrow head) and splenic infarct (arrow).

**Figure 2:**
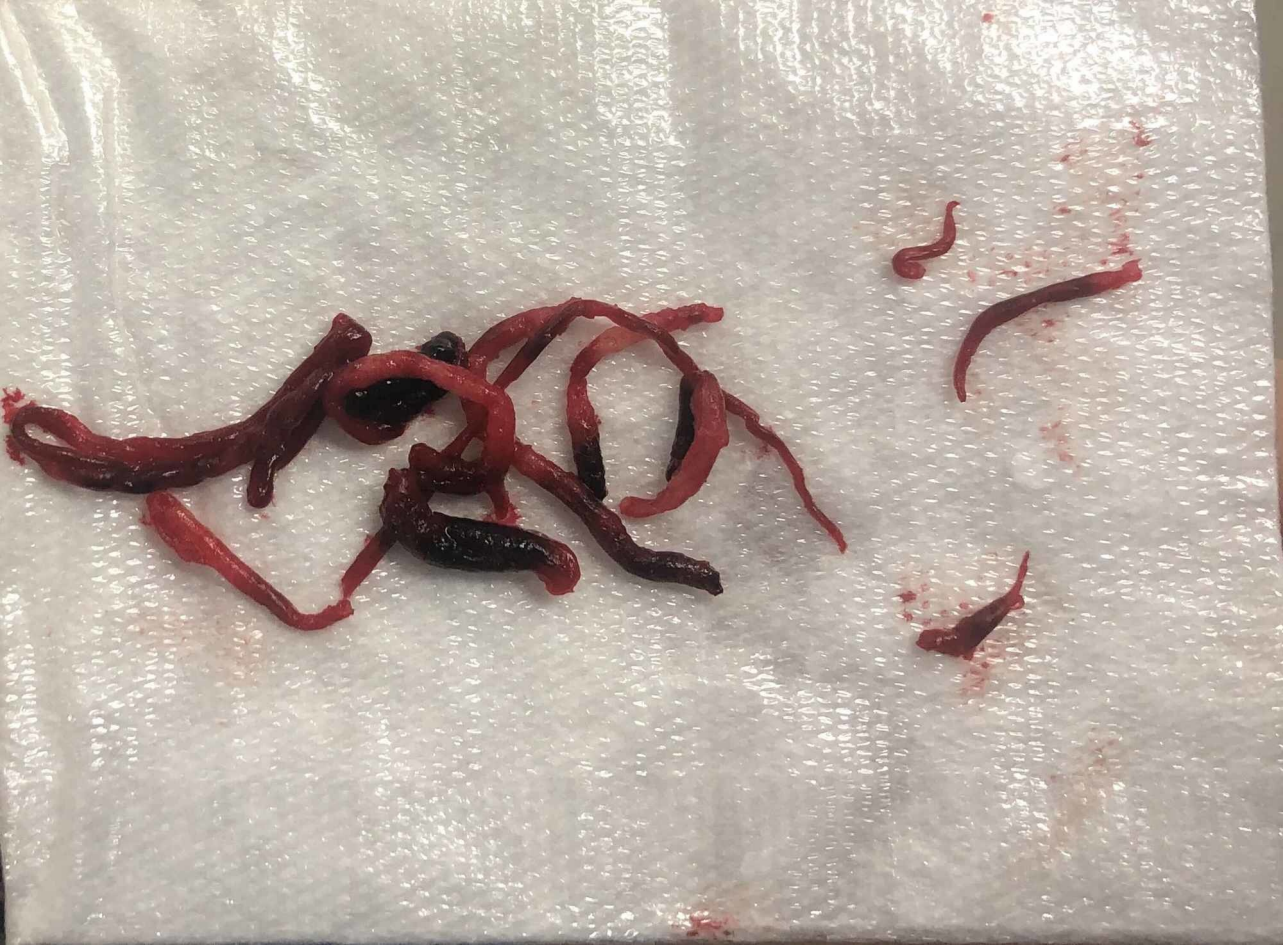
Intraoperative pictures of gelatinous thrombus removed from right leg tibial arteries.

A 69-year-old female with a history of essential hypertension presented to the hospital with pain and decreased sensation in the right foot. She was discharged from the hospital a week before this presentation, after recovering from a mild COVID-19 infection. On physical examination, her right foot appeared pale and cold. She had mottling of the skin and absent pulses distally on the right extremity. Labs revealed a D-dimer of 1799 ng/mL (reference range <231 ng/mL), and a platelet count of 600,000/microliter; her labs during the prior admission were notable for a D-dimer of 2750 ng/mL and a normal platelet count. She underwent computerized tomography angiography (CTA) that demonstrated thrombotic occlusion in all tibial arteries on the right leg along with intraluminal aortic thrombus in the visceral aorta, evidence of splenic infarcts , and thrombotic occlusion of the left distal popliteal artery with a clinically well-perfused left foot (Figure 1). The patient was started on IV heparin and subsequently underwent right popliteal and tibial thrombectomy. A large amount of acute and subacute thrombi was removed with the restoration of pulsatile flow to the foot (Figure 2). A decision was made not to intervene on the aortic thrombus, which was likely the source of her thromboembolism with plans to continue anticoagulation. She was discharged home on therapeutic doses of rivaroxaban, along with low dose aspirin.

 

Case 2

**Figure 3:**
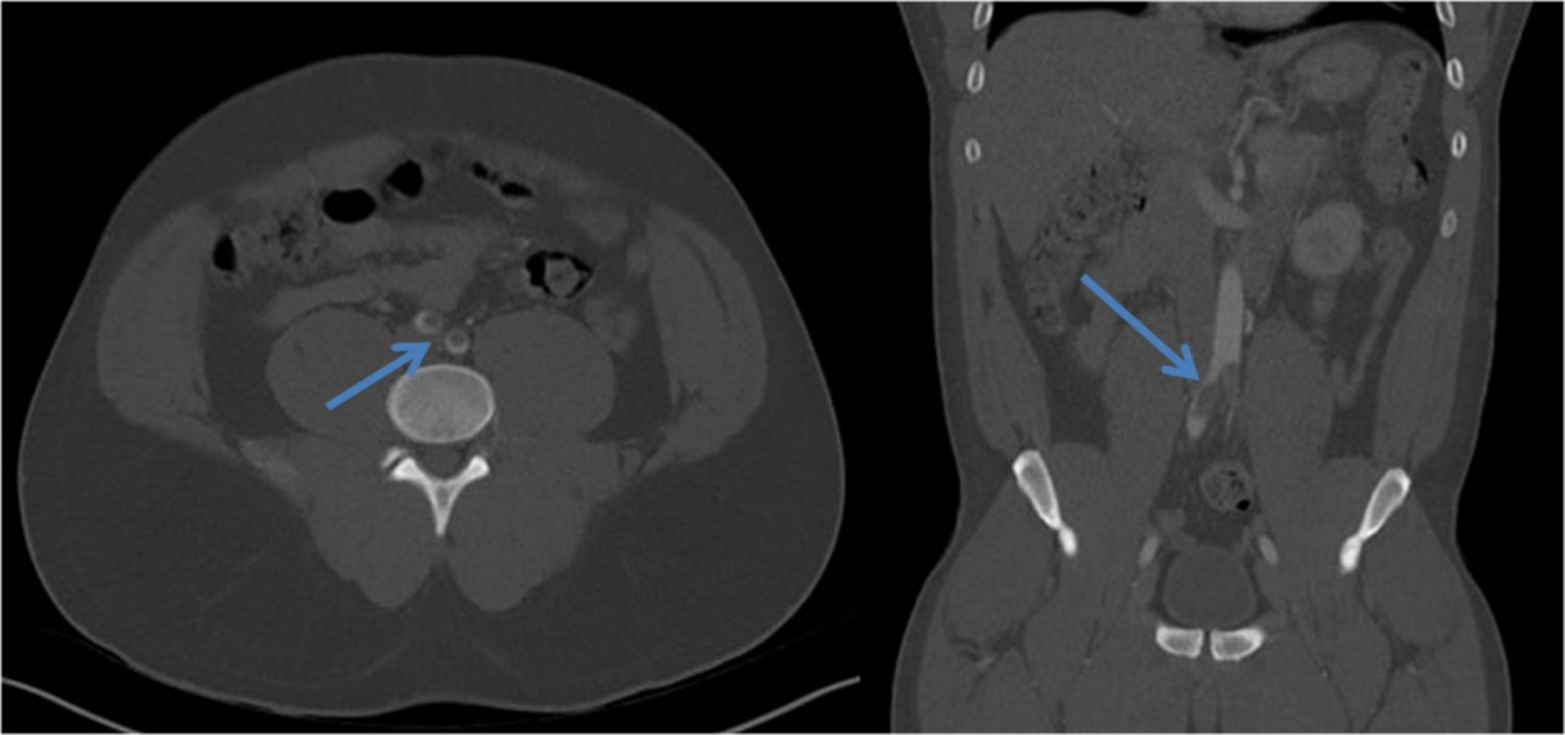
Thrombus at the aortic bifurcation with extension into both common iliac arteries (arrow).

A 33-year-old male with a history of essential hypertension presented to our hospital with fever and acute onset of right foot pain. He tested positive for COVID-19 infection. He was hemodynamically stable on room air. Physical examination was notable for the loss of bilateral femoral pulses. Labs revealed a normal complete blood count (CBC) and a D-dimer level of 493 ng/mL. He underwent a CTA abdominal aortic study that revealed an occlusive thrombus at the aortic bifurcation with near-complete occlusion of the right common iliac artery and significant stenosis of left common iliac artery (Figure 3). The patient had no atherosclerotic risk factors and thromboembolism was presumed to be COVID-19 related coagulopathy. The patient was started on IV heparin and subsequently underwent bilateral iliofemoral thrombectomy. He was discharged thereafter on therapeutic doses of rivaroxaban.

Case 3

**Figure 4:**
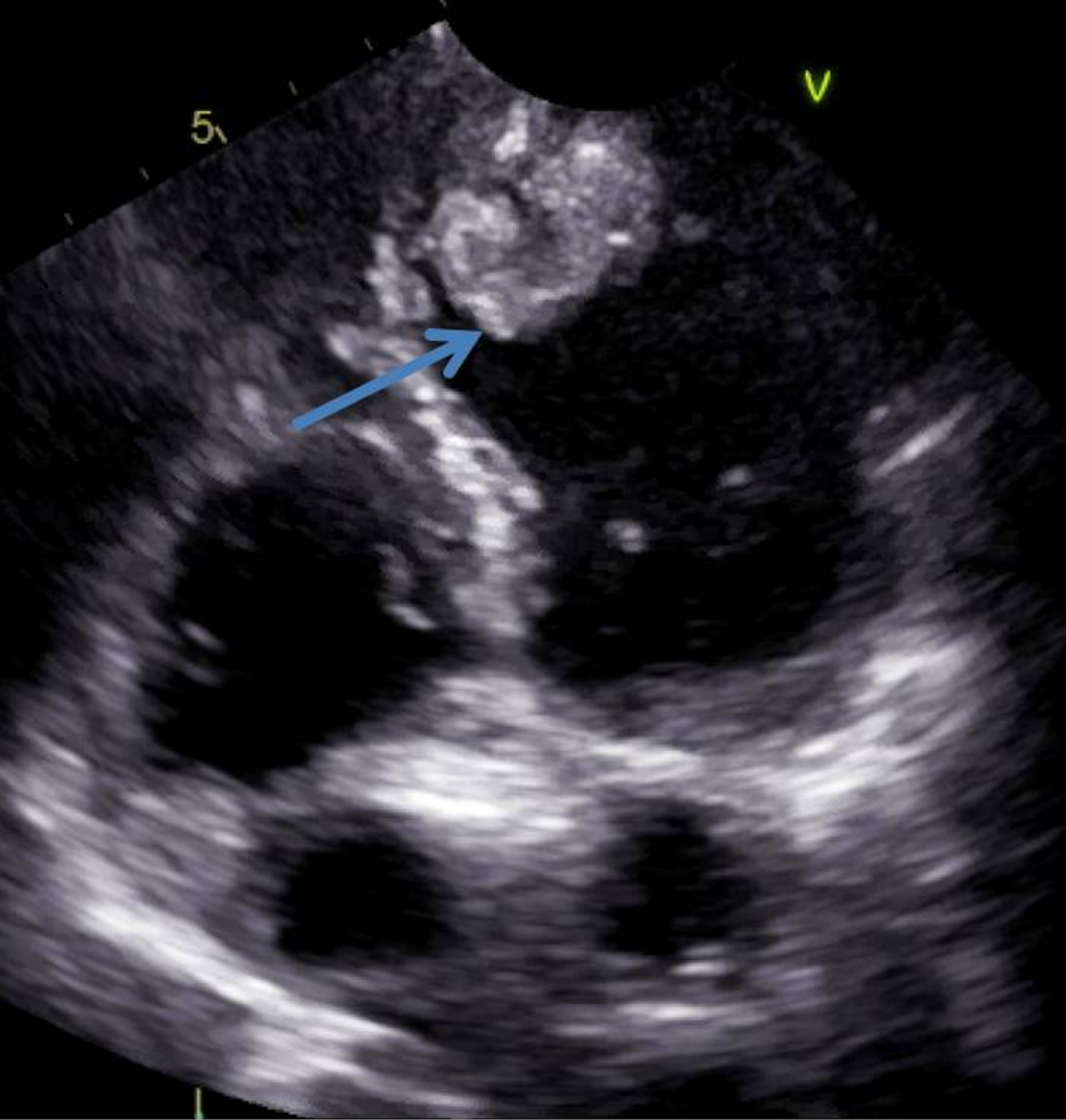
Echocardiogram -- apical four-chamber view demonstrating a heterogeneous left ventricular thrombus at the apex (arrow).

A 69-year-old female with a history of pulmonary embolism on apixaban presented to the hospital for alcohol detoxification. During her stay, she developed increased work of breathing. She underwent CTA of the chest which showed patchy bilateral ground-glass airspace opacities and subsequently tested positive for COVID-19. Labs revealed a normal CBC and a D-dimer of 4558 ng/mL. As the patient was on apixaban at home, she was switched to therapeutic low molecular weight heparin. Her hospital course was complicated by a new onset of significantly decreased ejection fraction of 25%-35% and evidence of a large left ventricular (LV) thrombus at the apex (Figure 4). Unfortunately, the patient developed a massive stroke in the territory of the left middle cerebral artery and clinically deteriorated. The family decided to withdraw care and keep the patient comfortable.

## Discussion

It is a well-established fact that severe sepsis is associated with the release of inflammatory cytokines such as interleukin (IL)-6, IL-8, tumor necrosis factor-alpha that promote activation of the coagulant pathway by increasing the expression of tissue factor. The activation of tissue factor on monocytes and endothelial cells leads to the initiation of the extrinsic coagulation cascade and the generation of thrombin [2]. Multiorgan dysfunction in COVID-19 is attributed to a complex interaction between thrombosis and inflammation, causing macro- and micro-thrombosis. 

Our patients who developed arterial thrombosis as a sequela of COVID-19 infection were relatively asymptomatic, and there was a low suspicion of prothrombotic state due to sepsis or acute respiratory distress syndrome. This suggests that COVID-19 independently induces a prothrombotic state regardless of other systemic complications and molecular pathways initiated by sepsis. Further studies are needed to elucidate the exact mechanism. Zhang et al. described a case of COVID-19 with multiple cerebral and limb infarcts in the presence of anti-cardiolipin IgA, anti-β2-glycoprotein IgA, and IgG antibodies [3]. The presence of antiphospholipid antibodies is found in association with several viruses such as Hepatitis C and HIV. However, it is unknown if their presence in COVID-19 may predispose to a clinically significant coagulopathy. 

Elevated D-dimer level is considered a prognostic factor [4-5]; however, it may underestimate the risk for thrombosis and clot burden. Although the role of low molecular weight heparin and direct oral anticoagulants in thromboprophylaxis of acutely ill medical patients at discharge has been studied, there is no current data about thromboprophylaxis of COVID-19 patients at discharge [6-7]. There are individual hospital-based protocols currently being utilized to start prophylactic anticoagulation based on the risk profile, especially with D-dimer > 2000 ng/mL to prevent venous thromboembolism (VTE). However, their role in preventing arterial thromboembolism is unknown. Risk stratification based on D-dimer levels may be misleading because an elevated D-dimer is just a marker of increased risk for thrombosis and not an absolute threshold. This is evidenced by the fact that our patients presented with varying levels of D-dimers, yet developed arterial thrombosis. The patient in Case 1 who developed an acute arterial thrombosis had a D-dimer of 1799 ng/mL. The patient in Case 2 developed a saddle aortic embolus with a D-dimer of <500 ng/mL, and the patient in Case 3 with LV thrombus had a D-dimer of 4558 ng/mL. An autopsy study of the patients affected by COVID-19 demonstrated an increased incidence of DVT in 58% of the patients, with one-third of the autopsies showing pulmonary embolism. Interestingly, most of these findings were not clinically suspected and only came to light after the autopsy [8]. These studies highlight the importance of thromboembolic prophylaxis in patients with COVID-19, independent of the severity of disease and the range of D-dimer levels. 

Although there are many reports of COVID-19 coagulopathy predisposing patients to VTE, there are only a few reports of arterial thrombosis leading to ischemic stroke and acute limb ischemia. A single-center in New York city observed five events of large vessel ischemic stroke in a two week period [9]. In Case 2, our patient developed a saddle occlusion of the aorta with an unremarkable echocardiogram and no evidence of underlying atherosclerosis, which to the best of our knowledge, is the second case of aortic thrombus. Le Berre et al. reported a case of a free-flowing aortic thrombosis without aortic atherosclerosis in a patient with COVID-19 [10]. The interesting aspect of our case is that the patient had a saddle occlusion of the aorta with loss of pulses in the distal extremities, which is very rare. 

## Conclusions

Thromboembolic prophylaxis for COVID-19 patients discharged from the hospital is now routinely used based on D-dimer levels. However, analyzing our cases, it might be beneficial to consider prophylactic anticoagulation for an extended time of two to four weeks based on clinical profile, rather than an absolute D-dimer level.

## References

[1] Connors JM, Levy JH (2020). Thromboinflammation and the hypercoagulability of COVID-19. J Thromb Haemost.

[2] Stouthard JM, Levi M, Hack CE (1996). Interleukin-6 stimulates coagulation, not fibrinolysis, in humans. Thromb Haemost.

[3] Zhang Y, Xiao M, Zhang S (2020). Coagulopathy and antiphospholipid antibodies in patients with Covid-19. N Engl J Med.

[4] Huang C, Wang Y, Li X (2020). Clinical features of patients infected with 2019 novel coronavirus in Wuhan, China. Lancet.

[5] Tang N, Li D, Wang X, Sun Z (2020). Abnormal coagulation parameters are associated with poor prognosis in patients with novel coronavirus pneumonia. J Thromb Haemost.

[6] Chi G, Gibson CM, Kalayci A (2019). Extended-duration betrixaban versus shorter-duration enoxaparin for venous thromboembolism prophylaxis in critically ill medical patients: an APEX trial substudy. Intensive Care Med.

[7] Spyropoulos AC, Lipardi C, Xu J (2019). Improved benefit risk profile of rivaroxaban in a subpopulation of the MAGELLAN study. Clin Appl Thromb Hemost.

[8] Wichmann D, Sperhake JP, Lutgehetmann M (2020). Autopsy findings and venous thromboembolism in patients with COVID- 19: a prospective cohort study. Ann Intern Med.

[9] Oxley TJ, Mocco J, Majidi S (2020). Large-vessel stroke as a presenting feature of Covid-19 in the young. N Engl J Med.

[10] Le Berre A, Marteau V, Emmerich J, Zins M (2020). Concomitant acute aortic thrombosis and pulmonary embolism complicating COVID-19 pneumonia. Diagn Interv Imaging.

